# Duodenal Adenocarcinoma in a Patient with Partial Intestinal Malrotation

**DOI:** 10.1089/pancan.2018.0005

**Published:** 2018-06-01

**Authors:** William T. Li, Sonia Sethi, Adrienne N. Christopher, Deepika Koganti, Charles J. Yeo

**Affiliations:** Department of Surgery, Jefferson Pancreas, Biliary, and Related Cancer Center, Thomas Jefferson University, Philadelphia, Pennsylvania.

**Keywords:** pancreaticoduodenectomy, intestinal malrotation, congenital anomaly

## Abstract

**Background:** Small bowel cancers, specifically duodenal cancer, occur at very low rates but require aggressive surgical resection when diagnosed. An even rarer finding is the presence of intestinal malrotation.

**Case Presentation:** We present the unique case of a patient with both duodenal cancer and partial intestinal malrotation undergoing pancreaticoduodenectomy. We discuss the challenges faced and techniques used to successfully perform a surgical resection in this circumstance.

**Conclusion:** Understanding of intestinal malrotation and review of the imaging is crucial in preparing for a resection of a duodenal tumor in a patient with this condition.

## Background

Small bowel neoplasms are a rare form of cancer in the United States, with ∼10,470 estimated new cases and 1450 estimated deaths per year.^1^ Although the small bowel comprises 91% of the total surface area of the alimentary tract, small bowel cancer comprises only 3.3% of estimated new cases of alimentary tract cancers per year.^2^ Another rare finding in adults is intestinal malrotation (IM). A review of cases at the Massachusetts General Hospital found only 82 confirmed diagnoses of IM in adults for a 17-year period.^3^ In adults, IM is often asymptomatic and found incidentally. We present a unique case of a 76-year-old male who presented with both duodenal cancer and incidental small bowel malrotation with associated radiological studies, pathology report, and a review of literature.

## Presentation of Case

**FIG. 1.**
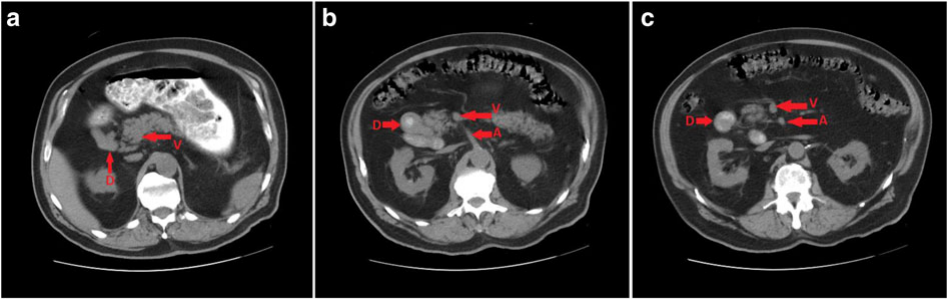
A CT scan without IV contrast showed generalized esophageal wall thickening and mild duodenal (marked by “D”) wall thickening **(b)**. The duodenal sweep does not cross the midline **(a–c)**, consistent with a malrotation. There is no obstruction identified. The transverse colon evaluation was limited due to motion blur. The SMV (marked by “V”) is anterior and to the right of the SMA (marked by “A”), as would be found normally. EUS performed showed a duodenal mass in the second portion of the duodenum that measured 3–4 cm. CT, computed tomography; EUS, endoscopic ultrasound; SMA, superior mesenteric artery; SMV, superior mesenteric vein.

The patient is a 76-year-old Caucasian male who initially presented with melanotic stools and anemia requiring multiple blood transfusions. He had a medical history of hypertension, hyperlipidemia, chronic kidney disease, and a myocardial infarction that resulted in a cardiac stent placement. During his workup for anemia, he underwent an esophagogastroduodenoscopy (EGD) and colonoscopy. His colonoscopy revealed no worrisome lesions in the colon. EGD revealed a large malignant appearing mass 4–5 cm in size just adjacent to the ampulla, which was biopsied. Pathology analysis returned as invasive moderately differentiated adenocarcinoma. He had a computed tomography to further delineate the mass and to assist with operative planning, which showed thickening of the duodenum as well as a partial malrotation at the third and fourth portions of the duodenum such that the ligament of Treitz failed to cross the midline (Fig. 1). Laboratories including serum tumor markers, carcinoembryonic antigen (CEA) and carbohydrate antigen 19-9 (CA 19-9), were within normal limits. Owing to his biopsy proven duodenal adenocarcinoma, its proximity to the pancreatic head, and the apparent structural anomalies, the patient was recommended to undergo surgical resection through a pancreaticoduodenectomy. His imaging was carefully reviewed preoperatively, and the partial malrotation was noted. The patient was prepared for his Whipple resection.

### Surgical findings

The patient was taken to the operating room for an open pancreaticoduodenectomy. Upon exploration, the duodenum and head of the pancreas revealed a palpable mass at the level of the second part of the duodenum and extending close to the first part of the duodenum and further caudal. It was necessary to proceed with a classic Whipple procedure since a pylorus-preserving pancreaticoduodenectomy would have compromised the duodenal margin. There was no evidence of carcinomatosis, malignant lesions in the liver, or omental implants. On exploration, various structures were abnormal. The duodenal C loop was vertical, and there was an ectopically located ligament of Treitz; it was found on the right side and inferior to its typical location. In addition, the presence of malrotation in the setting of significant mesenteric adipose tissue warranted that a longer portion of his jejunum be resected to yield sufficient mobility of the retained proximal jejunum to allow for a tension-free anastomosis of the jejunum to the pancreas remnant and biliary tree. The patient had normal positioning of the mesenteric arteries, which facilitated normal dissection of the pancreas around the superior mesenteric vein (SMV) and superior mesenteric artery (SMA). However, the presence of considerable mesenteric fat and bowel malrotation provided significant, although overcomable, challenges to our operation.

The pancreaticojejunostomy, hepaticojejunostomy, and gastrojejunostomy anastomoses were completed without incident. The specimen was sent off to pathology analysis and the frozen section analysis revealed a duodenal adenocarcinoma, with all resection margins being negative for malignancy.

### Postoperative care

Postoperatively, the patient experienced transient delirium and developed an International Study Group for Pancreatic Fistula (ISGPF) grade B pancreatic leak that was drained percutaneously. He was discharged to a rehabilitation facility on postoperative day 13.

### Pathological findings

The pancreaticoduodenectomy specimen contained a 3.2 × 2.5 × 2 cm exophytic tan pink to red firm granular mass within the duodenum, ∼13 cm from the proximal gastric resection margin. The mass was adjacent to a normal ampulla of Vater. Upon sectioning, the mass grossly appeared to extend into the muscularis propria but did not involve the underlying pancreatic parenchyma. There was no dilation of the pancreatic duct. The mass was a moderately differentiated adenocarcinoma of the duodenum with focal invasion of the subserosal fatty tissues. A microscopic neuroendocrine tumor was also identified within the duodenal wall. It was well differentiated, low grade, and 2 mm in greatest dimension. All 12 regional lymph nodes recovered were negative for metastatic disease. All resection margins were also negative for neoplasm. The final staging of the duodenal adenocarcinoma was T3N0M0.

### Molecular testing

A Pan-Cancer 42-Gene mutation panel of the specimen was performed. Analysis showed a G12V mutation of the KRAS gene, as well as a T>G nucleotide change in codon 560-3 of the TP53 gene. Both of these changes were classified as pathogenic. No further mutations were identified in the other 40 genes in the panel.

## Discussion and Literature Review

Cancer of the small bowel is a rare entity and accounts for only about 3.3% of gastrointestinal tract neoplasms. IM is a congenital anomaly that occurs in 1 in 500 live births and very rarely presents in adults.^4,5^ Small bowel cancer occurring in the setting of IM is a rare combination and can pose unique surgical challenges as the anatomical variations can often be located in the pancreatic region. Rittenhouse et al. performed a retrospective review of the incidence of gastrointestinal malrotation discovered incidentally on preoperative imaging before hepatobiliary surgery. They found an incidence of 3 out of 1220 cases, or 0.2%.^6^ In planning for these cases, the surgeon must be prepared to deviate from the classic surgical techniques utilized to ensure a safe resection.

For example, in two cases reported in Rittenhouse et al., the jejunal limb had to be brought up for the pancreaticojejunostomy and hepaticojejunostomy in a paracolic manner, as opposed to the standard retrocolic manner.^6^ In our case, we were required to divide the proximal jejunum 60 cm below the ligament of Treitz to create a tension-free anastomosis. This is a much longer proximal limb than is typically required. Mateo et al. discuss the need to modify the approach to pancreaticoduodenectomy secondary to the presence of Ladds' bands and the need to release these peritoneal extensions while exercising caution in avoiding the vascular arcades of the small bowel.^7^

It is also imperative to have an understanding of the vascular variants that may be present in a patient with malrotation. The presence of the SMV found to the left of the SMA, known as the SMV rotation sign, may be indicative of IM in the adult patient.^8^ Although not the case in our patient, Mateo et al. demonstrated significant vascular variation of the SMV, SMA, and celiac trunk in three patients with IM undergoing pancreaticoduodenectomy.^7^ These vascular structures deliver most of the blood supply to the pancreas and midgut and are the centerpieces for dissection during pancreaticoduodenectomy. The presence of abnormalities in these critical vessels must be closely scrutinized on preoperative imaging preoperatively to prevent unintentional injury or ligation of the vessels.

Finally, the ligament of Treitz can often be found in an abnormal position or may be missing altogether in patients with IM.^9–11^ As the ligament of Treitz is an important landmark for identification of the mesenteric vein during pancreaticoduodenectomy, absence or ectopic placement of this structure carries with it the possibility of intraoperative injury or postoperative complications. In our case, the ligament of Treitz was displaced from the normal location and somewhat inferior. We worked carefully along the plane between the uncinate process of the pancreas and the transverse mesocolon to identify and dissect the SMV.

## Conclusion

We present a rare case of a patient with adenocarcinoma of the duodenum and IM treated with pancreaticoduodenectomy. Close review of imaging and understanding of IM helped us prepare for the challenging but ultimately successful resection of the tumor.

## Abbreviations Used

EGD: esophagogastroduodenoscopy
IM: intestinal malrotation
SMA: superior mesenteric artery
SMV: superior mesenteric vein
